# Collaborative case-based learning with programmatic team-based assessment: a novel methodology for developing advanced skills in early-years medical students

**DOI:** 10.1186/s12909-022-03111-5

**Published:** 2022-02-07

**Authors:** Mariel James, Ana Madeira Teixeira Baptista, Deepak Barnabas, Agata Sadza, Susan Smith, Omar Usmani, Chris John

**Affiliations:** Faculty of Medicine, Imperial College School of Medicine , Hammersmith Campus, Room 6N5c, Commonwealth Building, W12 0NN London, UK

## Abstract

**Background:**

Imperial College London launched a new, spiral undergraduate medical curriculum in September 2019. Clinical & Scientific Integrative cases (CSI) is an innovative, flagship module, which uses pioneering methodology to provide early-years learning that [1] is patient-centred, [2] integrates clinical and scientific curriculum content, [3] develops advanced team-work skills and [4] provides engaging, student-driven learning. These aims are designed to produce medical graduates equipped to excel in a modern healthcare environment.

**Methods:**

CSI has adopted a novel educational approach which utilises contemporary digital resources to deliver a collaborative case-based learning (CBL) component, paired with a team-based learning (TBL) component that incorporates both learning and programmatic assessment. This paper serves to explore how first-year students experienced CSI in relation to its key aims, drawing upon quantitative and qualitative data from feedback surveys from CSI’s inaugural year. It provides a description and analysis of the module’s design, delivery, successes and challenges.

**Results:**

Our findings indicate that CSI has been extremely well-received and that the majority of students agree that it met its aims. Survey outputs indicate success in integrating multiple elements of the curriculum, developing an early holistic approach towards patients, expediting the development of important team-working skills, and delivering authentic and challenging clinical problems, which our students found highly relevant. Challenges have included supporting students to adapt to a student-driven, deep learning approach.

**Conclusions:**

First-year students appear to have adopted a patient-centred outlook, the ability to integrate knowledge from across the curriculum, an appreciation for other team members and the self-efficacy to collaboratively tackle challenging, authentic clinical problems. Ultimately, CSI’s innovative design is attractive and pertinent to the needs of modern medical students and ultimately, future doctors.

**Supplementary Information:**

The online version contains supplementary material available at 10.1186/s12909-022-03111-5.

## Background

Imperial College London’s School of Medicine launched a new, spiral undergraduate curriculum in 2019 with emphasis on patient-orientated integration of content, digital innovation, and student-driven, active learning. Medical schools are increasingly recognising the importance of integrating curriculum contents both vertically (across the years of the degree) and horizontally (between contemporaneous modules), to represent the integrated nature of a doctor’s role and to develop students’ abilities to apply knowledge in basic, clinical and social sciences to build holistic understanding of patients [1, 2]. Additionally, available medical knowledge remains an ever-expanding field, with resultant saturation of curricula [3]. Developing the ability to find, evaluate and utilise relevant information [4, 5] is therefore increasingly important. In response to these needs, a pioneering flagship module called Clinical & Scientific Integrative cases (CSI), was created. It creatively combines collaborative case-based learning (CBL) with programmatic assessment that uses a team-based learning (TBL) structure; an approach which has not been documented previously. This novel methodology provides potential for great benefit in the advancement of team-working skills, problem-solving capabilities and clinical application of knowledge in early-years students. CSI aims to encompass four key principles; [1] to be patient-focused, [2] to integrate clinical and scientific learning, [3] to develop advanced team-work and collaboration skills and [4] to provide student-driven learning that is motivating and engaging. In delivering such learning from the *beginning* of an undergraduate curriculum, we hope to cultivate graduates with the capacity to integrate multi-level aspects of health (and thus deliver patient-focused care), with good skills in working with colleagues and with the confidence to solve the clinical problems they will face as newly qualified doctors.

CBL “prepares students for clinical practice. It links theory to practice, through the application of knowledge to authentic cases, using inquiry-based learning” [6]. TBL combines team-work with investigation, use of resources and *application* of knowledge and is a valuable tool for improving conceptual understanding [7]. A recent increase in TBL usage reflects a shift towards developing skills that will equip graduates to thrive, rather than memorization of facts [8]. CBL and TBL are therefore both useful tools for contemporary curricula. In combination, we believe they have potential to profoundly impact the learning of early-years students. Particular features of our combined approach include a focus on collaborative work (shown to increase engagement and enhance student-driven learning [9]), professionally-produced patient videos that make learning feel authentic [10] and enhance student immersion, and a persisting central theme of a patient, serving as a focus to integrate learning. Perhaps most novel is our use of TBL in its full structure as a means of programmatic assessment. Whilst individual readiness assurance test (iRAT) and team readiness assurance test (tRAT) components (composed of single-best answer questions (SBAQs)) have been used for assessment previously [11–14], team *application* components have not. Collaborative testing may expedite the development of reliable team-working, critical thinking and problem-solving skills [15], support the development of clinical initiative and expertise, and provide impetus to engage in formative learning events.

The main aim of this study is to present the design and delivery of a pioneering and innovative module underpinned by four key principles (stated above) relevant to the needs of a modern medical curriculum. We will present students’ perspectives (collected via systematic surveys) about the different facets of the module, and analyse whether, from their experiences, the module can achieve these core principles. We will discuss these perspectives in conjunction with aspects of module design, programmatic assessment results and our own experience in order to critically review our novel approach. A secondary aim is to specifically explore the role of TBL-based programmatic assessment in student engagement and the development of key skills; namely the ability to integrate knowledge to create patient-centred management plans, and to work effectively in teams.

## Methods

### Module Design

 CSI was developed by a curriculum reform group, based on significant collective experience in higher education and review of the available literature on relevant teaching practices. Year one content was designed to reflect common presentations likely to be encountered during students’ first clinical placements and on this basis, fictional but authentic patient cases were created. A core module team was established, comprising of module leads (one clinical, one scientific), teaching fellows (one clinical, one scientific) and an instructional designer. Case content was discussed with experts and other module leads; important for the optimisation of an integrated, spiral curriculum design.

**Fig. 1:**
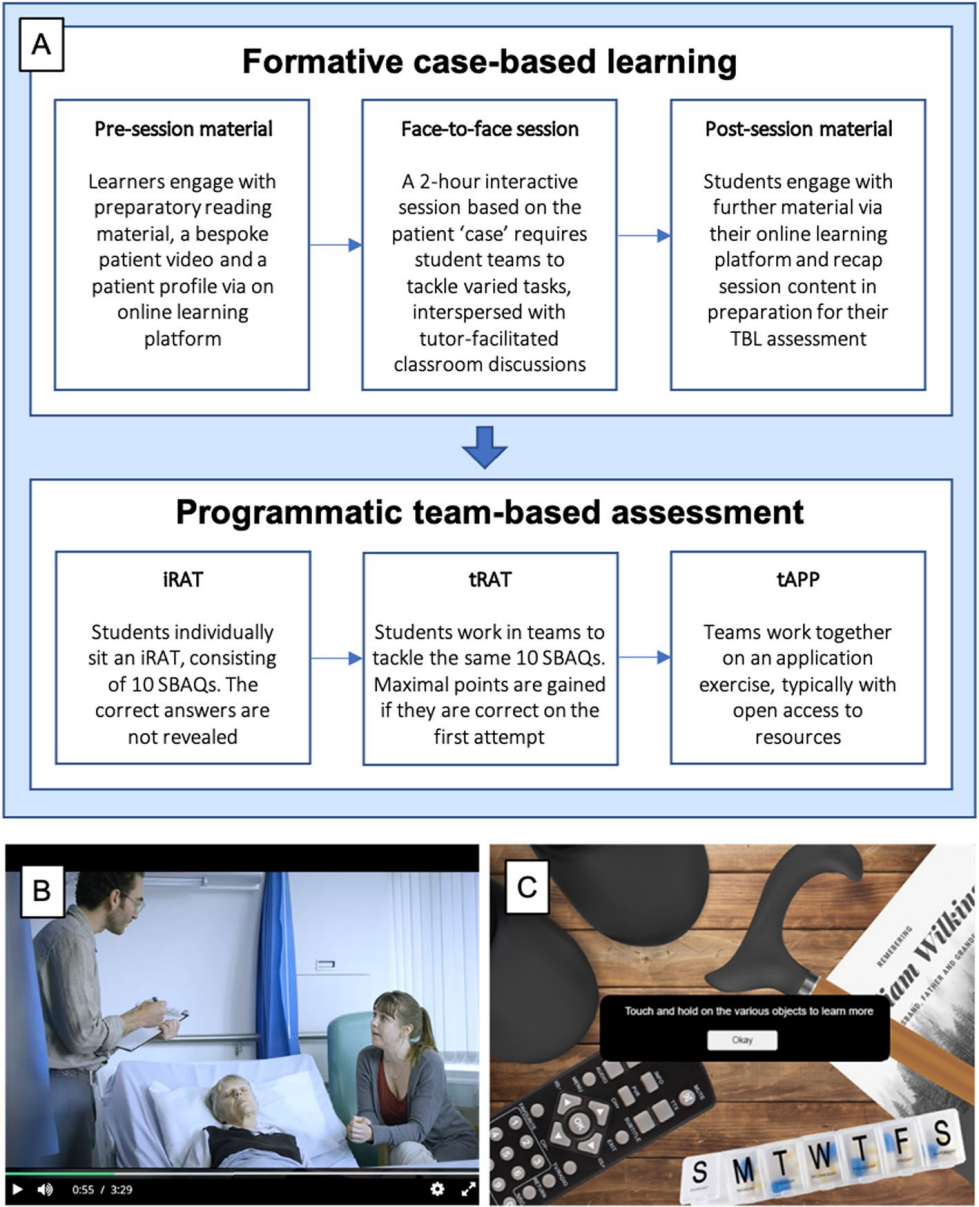
**A**) Structure of one CSI ‘case’. **B**) Screenshot from a ‘case’ video (left) and **C**) from an illustrative patient profile (right)

The core structure of CSI is shown in Fig. 1. Initially, ten CSI ‘cases’ were planned for year one. However, the 2020 COVID-19 pandemic necessitated remote delivery methods during term 3, therefore, this paper will focus solely on the first 6 cases (terms 1-2), delivered in the format initially intended.

### Case-based learning component

Asynchronous guided online material was released one week prior to each face-to-face session. The ‘patient’ was introduced to students using an illustrated character profile and a bespoke videographic representation of their consultation (Fig. 1). The profile required digital interaction with a virtual collection of objects, which provided important information about the patient (often referenced during sessions), helping to build a holistic understanding. The bespoke videos utilised original scripts, influenced by relevant clinical/communication specialists and created by a specialist digital team using professional actors. These introduced the clinical problem, whilst maintaining a degree of uncertainty, essential for exploration within the upcoming session. Additional pre-session material included a case introduction video from module leads and preparatory reading (bespoke content or reputable online resources) to ensure sufficient basic understanding.

Each 2-hour face-to-face CBL session was facilitated by a scientific and clinical tutor pair. Approximately 50 students per session worked in pre-defined groups of 5-6. Groups remained constant throughout the year to facilitate the development of team dynamic. CBL sessions explored three broad themes relevant to the case, each comprising of 2-4 tasks. For example, ‘Mrs Wilkins’ (Fig. 1), is an elderly lady who falls and breaks her hip. Themes/tasks explored [1] risk factors for falls and fracture (including the mechanostat theory and osteoporosis), [2] radiological identification, management and outcomes of hip fractures and [3] recognising and managing delirium. The diverse content of each case built to produce an integrated and holistic understanding of the subject, featuring a balance of clinical and scientific content and regular representation of other aspects of the curriculum.

Tasks required groups to collaboratively produce answers and usually to submit these to a digital ‘whiteboard’ using an online audience response tool. Task format varied; groups might be required to submit short answers to a word cloud, complete a matching exercise, cast votes, or rank responses in order of preference. Tasks also varied in complexity, requiring and developing a range of cognitive skills and spanning Bloom’s taxonomy levels [16]. Each task was consolidated with an inclusive, enriching classroom discussion facilitated by tutors, drawing upon submitted responses and engaging the whole class simultaneously. These scaffolded the introduction of new and challenging concepts, distributed learning and ensured objectives were met.

Following a session, further resources were released for independent study, including a de-briefing video from module leads, a task recap and further reading to build upon session content.

### Team-Based Learning component

Each face-to-face session was paired with a TBL-based assessment (TBL-A), as a means of programmatic assessment (summarised in Table 1). Each TBL-A contained an iRAT, a tRAT and a team application exercise (tAPP). Each component contributed to the case mark, each case mark contributed to the end-of-module mark, and the end-of module mark was weighted to 16.5% of each student’s end-of-year mark.

**Table 1:** Components of a TBL assessment

	i-RAT	t-RAT	t-APP
Format	10 SBAQs Approached individually Closed-book	10 SBAQs Approached in teams Closed-book	Variable (see text) Approached in teams Open-book
Time allocation	15 min	20 min	75 min
Scoring	1 point per correct answer 10 points available	4 / 1 / -2 / -5 points if correct on first / second / third / fourth attempts (respectively) 40 points available	Variable points available, usually in the range of 25-45, dependent on the nature of the exercises
Weighting	Adjusted to 60% of TBL-A	Adjusted to 20% of TBL-A	Adjusted to 20% of TBL-A
	***Overall CSI mark weighted to contribute 16.5% of final end-of-year marks***		

TBL-As were carried out under exam conditions, with students synchronously present in an exam hall, accessing a dedicated online platform from university-issued electronic devices. Students worked in their pre-defined teams, with a group leader submitting consensus responses. tAPPs were related to the index case content and their formats varied, including: data interpretation to guide selection of appropriate antibiotics, production of an infographic for healthcare workers on patient-centred care in sickle cell anaemia, and making/explaining clinical decisions around short case studies. A number of clinical specialists contributed to tAPP design to ensure relevance and accuracy. Whereas iRATs/tRATs were closed-book, tAPPs were either open-book (allowing access to notes and web browsers), or provided specific online resources for reference. iRAT/tRAT scores were calculated automatically by the software and tAPPs were double-marked by faculty according to robust mark schemes. Following each TBL-A, students were provided with their iRAT/tRAT marks and either group-specific or cohort-level feedback on the tAPP. Students were encouraged to submit challenges for SBAQs that remained unclear after the tRAT process, via email. These were addressed in correspondence distributed to all students.

### Evaluation Methodology

This is an exploratory and descriptive case study [17]. Its objectives are two-fold: to understand how learners experienced the CSI module in relation to its key principles, and to analyse whether programmatic TBL-based assessment contributed to an integrated and lived experience of those principles. This is a single case study about the CSI module, implemented at Imperial College London School of Medicine to first-year students starting their MBBS programme in 2019. It relies on multiple sources of evidence, which will be brought together for an integrated discussion and understanding of the innovative nature of CSI.

#### Learners’ perspectives

We delivered a number of optional, online surveys to students. These were developed over several weeks by members of the CSI team in collaboration with a designated evaluation and research team. Surveys A and B were developed to capture information on student experience alongside more logistical information to aid adjustment and improvement of the module in its early stages. Survey C was designed with a greater focus on understanding student development, and is part of a longitudinal research project that will look at the development of self-efficacy traits across students’ first three academic years (the duration of CSI). Survey C therefore incorporates modified elements of validated scales around teamwork and empathy [18, 19] and where an appropriate validated scale was not available, elements of a pilot study around integration of knowledge [20]. The use of Survey A for research was deemed exempt from requiring ethical approval by the Faculty’s Medical Education Ethics Committee (MEEC). The use of Surveys B and C and exam results was granted ethical approval (MEEC1920-181).

Survey A was piloted with a ‘warm-up’ case, which students undertook to become acquainted with the module structure prior to the first programmatic case. Survey B and C, which were offered as one-offs, were not piloted. Many survey items sought practical feedback (for example material, timings, constructive alignment) or feedback on other aspects not relevant to this paper (including remote delivery during the pandemic). For this paper we have drawn upon survey items relevant to CSI’s four key aims. The full surveys are included in Additional file 1.

Survey A was offered to students after each of the cases 1-6, as a feedback link at the end of the TBL-A (on the final page of the digital session content). Students that chose to participate accessed the link and undertook the survey before leaving the examination room. A series of statements (15 closed items, of which 4 are used in this analysis), invited responses on a six-point Likert scale of: ‘strongly agree’, ‘agree’, ‘somewhat agree’, ‘somewhat disagree’, ‘disagree’ and ‘strongly disagree’.

Survey B was offered to students once, after the final year one case (delivered remotely during term 3), as a feedback link on the final page of TBL-A content. It asked about overall experiences of the cases in both original and remote formats, using a series of statements (16 closed items, of which 7 used in this analysis) and the same Likert scale.

Survey C was offered to students once via email, after completion of case 6 (the final case delivered in its intended format). Students were invited to score statements (16 closed items, of which 7 used in this analysis) from 0 to 100, where 0 meant “I cannot do this at all” and 100 meant “I am highly certain I can do this”, indicating their perceived capability.

In addition to the above scales, all three surveys featured a small number of additional, optional open questions allowing free-text responses.

## Results

There were 361 year one students. The number of respondents per survey varied from 57 to 275 per case for survey A (median respondents 119). Survey B yielded 74 respondents and survey C yielded 97 respondents.

### Surveys A and B: Experiences of CSI

Responses from survey A (repeated after each case) and survey B (distributed after the final case) are combined in Table 2 in order to categorise feedback by the key principles of CSI. This represents the percentage of respondents selecting each Likert response. For survey A, data is presented in the format of *median* percentages and *median* number of respondents per case. The number of survey A respondents for each case varied from 57 to 275. Data for survey B is shown as *absolute* percentages of respondents per Likert option, as the survey was only collected once. There were 74 responses, although not all respondents answered every question. For all statements in surveys A and B pertaining positively to the core principles of CSI, the *minimum* percentage of students selecting agree/strongly agree was 50%. In contrast, the highest percentage selecting disagree/strongly disagree was never greater than 9%.

**Table 2:** Likert scale survey responses from Survey A (median values for the six case-by-case surveys) and Survey B (absolute values for the single end of year survey)

CSI Core Principle	Survey Item (survey of origin)	Percentage of respondents (%)						Responses (n)
		Strongly agree	Agree	Somewhat agree	Somewhat disagree	Disagree	Strongly Disagree	
Patient-centred learning	The cases encouraged me to relate to the patient at hand (survey B)	12.5	48.2	28.6	7.1	3.6	0.00	56
		**60.7**		35.7		3.6		
Integration of clinical and scientific content	The cases encouraged me to integrate knowledge and skills from different areas (survey B)	30.7	48.4	19.4	0.00	1.6	0.0	62
		**79.0**		19.4		1.6		
	The cases built knowledge that I’ll remember (survey B)	14.7	44.1	25.0	10.3	5.9	0.0	68
		**58.8**		35.3		5.9		
Team-work / collaboration	The cases resulted in in-depth discussion with my colleagues (survey B)	23.7	57.6	11.9	6.8	0.0	0.0	69
		**81.4**		18.6		0.0		
	The process of discussing an answer in a team had a positive impact on my learning* (survey A)	32.7	46.3	19.5	2.0	0.7	1.3	118
		**75.5**		23.4		2.1		
	I was able to participate and make my voice heard in the group activities* (survey A)	40.8	42.6	11.7	1.7	0.6	0.9	118
		**83.5**		13.4		1.8		
Motivating and engaging learning	I found the cases to be stimulating and engaging (survey B)	25.0	48.4	15.6	6.3	4.7	0.0	64
		**73.4**		21.9		4.7		
	The cases required me to take responsibility for my own learning (survey B)	26.7	46.7	18.3	6.7	1.7	0.00	60
		**73.3**		25.0		1.7		
	The tAPP was stimulating and interesting* (survey A)	25.4	29.9	26.2	9.1	5.3	4.0	118
		**56.2**		36.1		9.2		
	This case motivated me to explore and learn more about this topic* (survey A)	15.1	35.3	32.1	9.7	4.9	2.2	118
		**50.1**		40.5		7.3		
	The face-to-face sessions provided clarity around the key learning from the tasks (survey B)	16.1	46.4	16.1	12.5	8.9	0.0	56
		**62.5**		28.6		8.9		

For items taken from survey A (repeated after each case), median percentages and respondents per case are shown. For items taken from survey B (distributed once), absolute percentages and respondents are shown

### Survey C: Development of self-efficacy

Responses are presented in the format of median student score per item. 97 students completed the survey, although only 89 students answered all questions. After six CSI cases, students’ responses indicated a high level of self-efficacy in relation to the key principles of CSI, as detailed in Table 3. A selection of relevant free text comments from this survey is included in Additional file 2.

**Table 3:** Self-efficacy scale responses from Survey C, following six on-campus cases

*CSI Core Principle*	*Survey Item*	Median response (scale of 0-100)	Interquartile range	Responses (n)
Patient-centred learning	*I can focus on individual patients in a holistic manner, incorporating elements of clinical and scientific significance*	**74.5**	13.5	90
	*I can apply the skills developed in CSI to evaluate other patient cases*	**75.0**	18.8	93
Integration of clinical and scientific content	*I can use clinical scenarios to achieve a deeper understanding of the basic science principles I have learned*	**79.0**	22.0	97
	*I can apply my understanding of basic science principles to clinical problems in order to contribute to better patient care*	**77.5**	18.75	90
	*I can explain why clinical and basic science integrated teaching is important to my development as a doctor*	**87.0**	18.0	92
Team-work / collaboration	*I can work with my team to achieve the goals we are set*	**82.0**	19.0	89
Motivating and engaging learning	*I can find the information/resources needed for our team to do our job well*	**74.0**	19.5	94

### Programmatic assessment performance

TBL-As results are summarised in Table 4. Of 361 first-year students, 6 were excluded from analysis due to interruption of studies. Mean scores per case were calculated from students in attendance. Although the iRAT and tRAT have different scoring systems, an increment from one to the other is typical; we observed increases ranging from 10.6 to 16.1% points across the six cases, with a median increment of 14.1 per case (IQR 2.1).

**Table 4:** Mean TBL-A scores across cases 1-6, divided by component

TBL-A	Students (n)	iRAT score		tRAT score		Mean iRAT to mean tRAT increment	tAPP score		Combined score	
		Mean (%)	SD	Mean (%)	SD		Mean (%)	SD	Mean (%)	SD
**Case 1**	348	74.6	13.1	85.2	10.7	10.6	61.7	12.9	74.1	9.0
**Case 2**	349	71.0	13.9	84.5	10.2	13.5	64.6	12.1	72.4	9.4
**Case 3**	343	50.6	16.6	65.7	17.7	15.1	59.7	10.2	55.5	12.1
**Case 4**	349	75.6	14.7	91.7	10.5	16.1	63.0	13.1	76.3	10.3
**Case 5**	350	63.7	14.0	76.4	12.5	12.7	66.4	7.9	66.8	9.6
**Case 6**	350	82.4	13.9	97.1	6.1	14.7	60.7	9.5	81.0	9.1
**Cases 1 to 6 (total)**	2089	69.7	17.6	83.5	15.6	13.8	62.7	11.3	71.1	12.8

Of six TBL-As, 88.7% of students attended six, 11.0% attended five and 0.3% attended four or fewer.

## Discussion

### Patient-focused learning

Patients want medical care that explores their concerns, seeks an integrated understanding of their world and provides management options that are mutually agreeable and enhance a continuing relationship with their doctor [21]. Such care is emphasised within the General Medical Council’s outcomes for graduates [22], indicating that medical education should develop the adoption of a flexible and empathetic approach towards patients. For this to become habitual, medical education must develop a drive to manage health in *partnership* with patients from the very beginning of training. Patient-centred medical education can therefore be described as being “about the patients, with the patients, and for the patients” [23].

CSI sessions were designed to maintain focus on the index ‘case’. For example, in a task on falls risk factors in Mrs Wilkins’ session, students were asked to highlight the risk factors most relevant to her. Other tasks were more transparent in building a patient-centred approach; for example, asking students to consider the ideas, concerns and expectations of a patient with sickle cell anaemia. The utility of such tasks in engendering a holistic approach has been evident not only from the thoughtful responses that we observed but also from feedback. A median of 89% of students (across six cases) at least somewhat agreed that the cases encouraged them to relate to the patient at hand (survey A, Table 2) and after six cases, the students gave a median confidence score of 74/100 for being able to focus on a patient in a holistic manner (survey C, Table 3). We received numerous comments across surveys about how CSI has taught students to appreciate different experiences of disease and to adopt a personalised approach. Collectively, this suggests that our first-year students have developed an understanding of the centrality of the patient to providing good medical care. We *only* received positive feedback on the use of patient cases to enhance learning, which indicates that these were both accessible and helpful.

CBL is integral to our patient-centred format. The video resources were specifically highlighted by students as helping to show how patients can be affected by disease. When delivered to qualified doctors, CBL has improved patient outcomes [24]; after taking part in CBL around diabetes management, physicians saw improved glycaemic parameters in their diabetic patients [25]. One of our early CSI case videos featured a doctor asking the patient to score their pain severity. We later obtained feedback from faculty that students had asked similar questions to patients during clinical placements (reporting learning this in CSI). Our students put patient-centred approaches into practice not only during formative tasks, but within TBL-As. One tAPP required students to read fictional case studies of patients attending a falls clinic, and using a number of resources, propose personalised management plans. Groups produced a huge variety of holistic suggestions, including home alterations, family support, medication changes and exploring the patients’ wishes. We have been consistently impressed by the insightful application of knowledge to patient-orientated tasks from these relatively inexperienced students.

### Integration of clinical and scientific content

Integration in medical education is thought to be highly beneficial. It supports students to draw connections between scientific, social, clinical, professional and personal parameters and in this way can be considered crucial in preparing students for the complex nature of their future roles [1]. It is also a key aspect of deep learning, the approach broadly understood to be most valuable in medical graduates [26] and encouraged by CSI. Integration may also result in enjoyable learning and increased student satisfaction [27].

We were interested in whether [a] students felt CSI was *successful* in integrating clinical and scientific concepts, and [b] whether they felt that this was *beneficial* to their learning. Almost all students (98.4%) at least ‘somewhat agreed’ that CSI “encouraged [them] to integrate knowledge and skills from different areas”, with 79% agreeing/strongly agreeing (survey B, Table 2). After six cases, students gave median confidence scores of 76/100 for being able to apply understanding of basic science to clinical problems in order to contribute to better patient care, and 85/100 for being able to explain *why* integrated teaching is important to their development (survey C, Table 3); this indicates that students feel integration will improve their clinical proficiency and also that after six cases, they have self-efficacy in their ability to draw upon this. Throughout the surveys, students provided comments on the perceived benefits of integration; they expressed that CSI connected their scientific learning with clinical application and helped them to understand the importance of other aspects of their curriculum. Although we didn’t receive any negative feedback about the integration of clinical and scientific content, not all students found this aspect easy, with one commenting that “it is hard to combine the two, however the more we do it the more we understand how to do so”, Another student said they felt more able to draw such connections independently as the year progressed (Additional file 2), indicating that CSI helped them to develop their own deep, integrative learning approach.

### Motivating and engaging learning

Active learning methodologies require individuals to participate and take responsibility for their learning. They allow learners to engage with material in a way that encourages discussion and critical thinking, and to build on pre-existing knowledge [28]. In this way, such methodologies lend themselves to integration of content and better understanding. Active learning can also develop communication skills by allowing students to practice reasoning and debating. A student-centred approach is thought to improve engagement [29] and it follows that active learning has the potential to develop the self-motivation that is crucial for a career that requires lifelong learning [28].

We observed a high level of engagement with CSI and believe that the reasons for this are two-fold. The first is its patient-orientated, integrative, active-learning format, described by students as interesting, engaging, relevant and fun (Additional file 2). This student-centred focus is tangible; 92% of respondents at least somewhat agreed that the cases required them to take responsibility for their own learning (survey B, Table 2). Although we did not enquire how students felt about this, we also observed that 82% of respondents per case at least somewhat agreed that they had been motivated to explore more about the topic (survey A, Table 2), from which one might interpret some enthusiasm. 84% of students at least somewhat agreed that the cases helped them to build knowledge they will remember (survey B, Table 2). We interpret these as rewarding figures given the volume of material that early students must cover and that a student-driven approach may be new to many first-year students [30].

The second contributor may lie in CSI’s assessment – an extrinsic driver to engage with formative sessions. It has previously been found that iRAT scores correlate with final examination scores when they contribute to grades, but are lower and do not correlate if they don’t [12, 14], suggesting that a summative test improves motivation to learn. We have experienced this directly, with attendance rates of ≥90% at face-to-face sessions and ≥96% at TBL-As. Being a new module, our first-year students have not been passed down informal resources from more senior students, a key element of a medical school’s hidden curriculum [31]. It will be interesting to observe what role this will play in future years. We intend to make yearly small changes to content in order to retain uncertainty, and we hope that the assessment element will continue to be a protective factor in maintaining engagement.

### Team-work and collaboration

The ability to work effectively in a team is a universally-accepted skill required of a medical graduate. Doctors must know how to build teams and maintain effective teamwork, identify the impact of their behaviour on others, work effectively with colleagues in ways that best serve patients, apply adaptability and a problem-solving approach to shared decision-making and recognise and respect the roles of others [22].

CSI has teamwork at its core and this clearly benefits learning. 81% of respondents agreed/strongly agreed that the cases resulted in in-depth discussions with colleagues (survey B, Table 2) and 75% of students agreed/strongly agreed that discussing an answer aided their learning, with 95% at least ‘somewhat agreeing’ (survey A, Table 2). A particularly novel aspect of CSI is the contribution of a tAPP to grades. There is little research into collaborative testing for high-stakes exams but there are clear benefits to be obtained, which our survey results support. Firstly, collaborative testing may improve academic performance and knowledge retention [32]. Secondly, it may improve communication and team-work [33]. Feedback from the earliest cases suggest some challenges with team dynamics (“we need to work on time management, and try and share out the tasks equally” and “all my efforts in trying to contribute have been dismissed”). However, in later surveys, students provided numerous comments on the benefits of CSI in developing teamwork skills. We posit that this learning curve may not have been as remarkable had there not been the extrinsic motivation of an assessment - supported by comments like, “time pressure means we’re all willing to communicate effectively, so has greatly increase[d] this skill” (Additional file 2). Thirdly, collaborative testing may result in learning about oneself, others and interpersonal dynamics [34]. Many of the students’ comments around CSI improving understanding of others’ perspectives are relevant not only to patients but to team-members. As such, CSI may help to develop empathy for colleagues, an important characteristic in interprofessional collaboration [35]. Several students commented on how CSI has improved relationships within the group, increased respect for others’ opinions and allowed students to realise when others need support. Finally, TBL may help to develop an understanding of the *value* of teamwork; many students commented on learning to appreciate/utilise the individual strengths of colleagues. Although early comments indicated some students did not feel able to contribute, later surveys featured more positive feedback. Taking an overview, 84% of respondents per case agreed/strongly agreed that they felt able to make their voice heard (survey A, Table 2) and after six cases, students gave a median score of 80/100 for how able they felt to work with their team to achieve the required goals (survey C, Table 3), indicating a good degree of perceived ability to work effectively with others.

#### Teamwork as a support structure for challenging tasks

The scaffolding that comes from a group structure and provision of resources allowed us to deliver complex tasks and tAPPs (featuring challenges authentic to those that a junior doctor might face), to first-year students. These provided experiential learning around team decision-making and problem-solving. A median of 77% of students per case at least ‘somewhat agreed’ that the tAPPs were stimulating and interesting - a pleasing outcome, given that assessments are not always enjoyed by students [36] (survey A, Table 2). This value reached as high as 95% for individual tAPPs, supported by comments such as: “the tAPP was really interesting; the combination of the medical history with the use of the British National Formulary made it feel very similar to how I perceive clinical practice to be”, and “I like that we get to test our knowledge in ways that we will actually use in the future” (Additional file 2). CSI aims to capitalise upon the benefits in *learning* that can come from assessment [37]; not only relevant to conceptual knowledge, but also the skills and experiences that the TBL-As involve, and this benefit appears to have been felt by students.

### Challenges and future steps

As a new module, CSI has experienced challenges. One has been in supporting students to adjust to a self-driven, deep learning approach. First-year students arrive with differing previous educational experiences, some of which will have favoured surface or strategic learning [26]; indeed, medical students may typically not shift to a deep learning approach until clinical years [38]. CSI favours the latter, and places emphasis on experiential learning through tasks that are semi-authentic mimics of future experiences in clinical teams, supported by consolidative tutor-facilitated discussions. However, it quickly became clear that more support was needed, as early sessions yielded requests for slides/recordings, with feedback such as “no slides were provided and didn’t know what I was supposed to make notes on”. Students also demonstrated pre-occupation with knowing what content might feature in the TBL-As and concern at the discrepancy of discussion points between different classrooms (“more guidelines would be helpful as different groups were taught different things”) (Additional file 2), in-keeping with the strategic approach that is common in medical students [26]. Student groups were permitted to submit ‘question challenges’, if they felt iRAT questions were unfair. A panel of academics from both the CSI team and the Year 1 assessments team met to discuss each challenge, approved or rejected the challenge and provided justification for the decision. This information was subsequently released to the students.

In response to feedback, we made key resource slides available after sessions and increased the detail in the post-session summaries, with positive feedback from students, who felt more able to immerse themselves and less worried about taking notes. We also provided tutors with clear learning points for each discussion. These measures aimed to provide better framework for revision whilst maintaining a focus on student-driven learning. Although we have no earlier data for comparison, after all cases, 79% of students at least somewhat agreed that the face-to-face sessions provided clarity around key learning points (survey B, Table 2). This issue could perhaps be tackled further with improved communications to students around the concepts of the module, for example ‘selling’ the advantages of experiential group-learning. It may also benefit students to understand that the focus of the TBL-As is on the associated *learning*, rather than to fail students. In our inaugural year, no student scored <50% in CSI which may reflect the support that a team-structure can provide. We also note the early feedback indicating difficulties experienced around team-working and collaborative assessment. We plan to discuss these issues pre-emptively in future introductory sessions, to normalise them as part of a learning curve, and encourage students to talk about any problems constructively within their team. Other possibilities include providing resources and escalation pathways for common team-work issues.

Obtaining a broad representation of our student cohort through survey responses was a challenge and is a possible limitation of this study. We observed a wide range of response rates to Survey A, from 16% (case 3) to 76% (case 2) of the year group. This may be for a variety of reasons: the verbal encouragement of the facilitator to provide feedback, whether the session had run late, students following others’ examples, or logistical aspects; for example, case 3 TBL-A was the students’ final activity prior to their Christmas break and students were therefore impatient to leave for the holidays. Surveys B and C captured 20% and 26% of the year group, respectively. Survey B was offered as on online link at the end of a remotely-delivered online session, making it easy for students to leave anonymously prior to completing it. Survey C was offered via email and it can be notoriously difficult to obtain responses this way without verbal encouragement. Obtaining high yield from online surveys is challenging across student and medical cohorts [39] for a number of reasons, and response rates may be lower for online surveys than other formats [40]. It is not uncommon for web-surveys conducted among students to yield response rates of <20% and it has been suggested that even response rates of 10% or less may be trusted provided the quality is checked [41] (although we acknowledge that low response rates may increase bias). A major contributor to low survey yield may have been ‘survey fatigue’; our students were faced with numerous, regular surveys from many modules due to the new curriculum changes. Although it is possible to improve uptake for online surveys with reminders, this was not considered appropriate given the volume of surveys being administered. Therefore, we had only brief windows to obtain responses. We made careful considerations to maximise responses [42]: limiting the number of items, making surveys clear and user-friendly, offering *optional* free-text spaces, and ensuring with regular bi-directional communication that students could see their feedback being addressed. Whilst we acknowledge the survey response rates could be higher, we feel the responses do overall demonstrate the experiences of students that we witnessed in leading the sessions. We also obtained verbal feedback during meetings with student representatives and feel that the survey responses reflect the feelings of the cohort that were communicated to us.

The composition of regular SBAQs based on new content was challenging. With content now established, it will be possible to write SBAQs further in advance, allowing for standard setting. Our six iRATs produced a wide range of mean scores (50.6-82.4%), demonstrating that it is feasible to write challenging SBAQs but also that providing sufficient challenge can be difficult. A difficult iRAT allows scope for substantial learning from the tRAT process (provided there is sufficient combined knowledge within the group to yield meaningful discussion). Conversely, if questions are too easy and understanding is universal, then tRAT discussion adds little. Interestingly, the iRAT to tRAT increment was reasonably stable across our TBL-As (10.6-16.1% points, Table 4). Even our poorest-scoring iRAT incremented by 15.1% points to tRAT, indicating that it was not *so* difficult that it limited the benefits to be had from discussion. However, this must be balanced with the fact that regular scores in this vicinity would result in a significant proportion of students failing the module. Therefore, we propose that aiming for a mean iRAT score of 60-80% allows both learning benefit and fair assessment.

## Conclusions

In conclusion, CSI has successfully incorporated a number of highly important and relevant educational objectives (both skills-based and knowledge-based) into one module by means of a novel structure that draws upon the most valuable elements of CBL and TBL. This structure appears to have helped first-year medical students to develop a patient-centred, holistic approach to care, to integrate knowledge and skills from across the curriculum, to develop an inquisitive and self-driven approach to learning and to build important skills in communicating and working with colleagues, as well as a sense of appreciation for what others in a team have to offer. These are all skills that may not previously have been addressed until much later in a medical curriculum. The design of CSI has also supported the delivery of authentic clinical problems, which our early-years students have embraced, successfully tackled and found both enjoyable and relevant. By featuring a patient as a central focus and placing students in teams, CSI begins to develop the advanced skills required of medical graduates from the very first day of medical school.

## Supplementary Information

**Additional file 1. **

**Additional file 2.**

## Data Availability

The datasets used and/or analysed during the current study are available from the corresponding author on reasonable request.

## Abbreviations

CSI: Clinical & Scienctific Integrative Cases
CBL: Case-based learning
TBL: Team-based learning
TBL-A: TBL-based assessment
iRAT: Individual readiness assurance test
tRAT: Team readiness assurance test
tAPP: Team application exercise
SBAQ: Single best answer question
