# Supplementary material for: Collaborative case-based learning with programmatic team-based assessment: a novel methodology for developing advanced skills in early-years medical students
Source: BMC Med Educ. 2022 Feb 7;22:81. doi: 10.1186/s12909-022-03111-5 (PMC8818362; doi:10.1186/s12909-022-03111-5)
Supplement: Supplementary file 1 — Additional file 1. [file 12909_2022_3111_MOESM1_ESM.docx]

# Additional File 1

## SURVEY A (delivered using an online survey tool after completion of each case (for cases 1-6))

**Items utilised in this paper are demarcated in bold**

*For the first 15 items, please grade each statement according to the identified scale (‘strongly agree’, ‘agree’, ‘somewhat agree’, ‘somewhat disagree’, ‘disagree’ and ‘strongly disagree’)*

1. I feel that the pre-session reading provided enough background information to prepare me for the face to face session
2. I feel that the content and tasks for the face-to-face session were pitched at the right level of difficulty

2a. You mentioned that you did not find the content and tasks pitched at the right level of difficulty. This was because...

- It was too difficult
- It was too easy

1. I found this case beneficial for integrating old and new knowledge from different sources (i.e. Principles of Medicine, Bioregulatory Systems, Lifestyle Medicine and Prevention, Professional Values and Behaviours etc)
2. I feel that the face-to-face session allowed enough time to cover the key material in the defined tasks
3. I feel that the post-session reading was relevant and built on material covered in the face-to-face session
4. I feel that the iRAT/tRAT questions for the team-based learning assessment were pitched at the right level of difficulty

6a. You mentioned that you did not find the iRAT/tRAT pitched at the right level of difficulty. This was because...

- It was too difficult
- It was too easy

1. I feel that the iRAT/tRAT questions checked understanding of the key themes introduced in the face-to-face session and reading
2. I feel that the tAPP for the team-based learning assessment was pitched at the right level of difficulty

8a. You mentioned that you did not find the iRAT/tRAT pitched at the right level of difficulty. This was because...

- It was too difficult
- It was too easy

1. **I feel that the tAPP exercise was stimulating and interesting**
2. **I feel that I was able to participate and make my voice heard in the group activities**
3. **feel the process of discussing an answer in a team had a positive impact on my learning**
4. **I feel that this case motivated me to explore and learn more about this topic**
5. At this point in time, I feel confident that I can produce an answer, using the CSI process of combining existing knowledge with other tools (i.e. research papers, guidelines, group discussion)
6. I feel stressed when faced with a question or task to which I don’t immediately know the solution
7. At the completion of this case, I feel confident in the knowledge I have gained

*For items 16 to 21, please answer as openly and honestly as you can (free text)*

1. Any comments on the pre-session reading material?
2. Any comments on the face-to-face session?
3. Any comments on the post-session reading material?
4. Any comments on the Team-Based Learning assessment session?
5. Any comments on your group or group work?
6. Any comments on your tutors?

## SURVEY B (delivered using an online survey tool after completion of *all* year one cases (ie, after the additional two term-3 cases delivered remotely during the Covid-19 pandemic))

**Items utilised in this paper are demarcated in bold**

*For the first 16 items, please grade each statement according to the identified scale (‘strongly agree’, ‘agree’, ‘somewhat agree’, ‘somewhat disagree’, ‘disagree’ and ‘strongly disagree’)*

1. **I found this format to build knowledge that I’ll remember: Term 1 and 2 (face-to-face) cases**
2. I found this format to build knowledge that I’ll remember: Term 3 (remote) cases
3. **I found this format to be stimulating and engaging: Term 1 and 2 (face-to-face) cases**
4. I found this format to be stimulating and engaging: Term 3 (remote) cases
5. **I found this format encouraged me to integrate knowledge from different areas: Term 1 and 2 (face-to-face) cases**
6. I found this format encouraged me to integrate knowledge from different areas: Term 3 (remote) cases
7. **I found this format resulted in in-depth discussion with my colleagues: Term 1 and 2 (face-to-face) cases**
8. I found this format resulted in in-depth discussion with my colleagues: Term 3 (remote) cases
9. **I found this format provided clarity around the key learning from the tasks: Term 1 and 2 (face-to-face) cases**
10. I found this format provided clarity around the key learning from the tasks: Term 3 (remote) cases
11. **I found this format required me to take responsibility for my own learning: Term 1 and 2 (face-to-face) cases**
12. I found this format required me to take responsibility for my own learning: Term 3 (remote) cases
13. **I found this format encouraged me to relate to the patient at hand: Term 1 and 2 (face-to-face) cases**
14. I found this format encouraged me to relate to the patient at hand: Term 3 (remote) cases
15. I found the visual content (slides / illustrations / videos) within this format to aid my learning: Term 1 and 2 (face-to-face) cases
16. I found the visual content (slides / illustrations / videos) within this format to aid my learning: Term 3 (remote) cases

*For items 17 to 19, please answer as openly and honestly as you can (free text)*

1. What were the positive impacts of remote delivery (lockdown) on your learning experience and approach in relation to CSI?
2. What were the negative impacts of remote delivery (lockdown) on your learning experience and approach in relation to CSI?
3. Any comments on the different delivery formats (terms 1&2 versus term 3)?

## SURVEY C (delivered using an online survey tool after the completion of case 6)

**Items utilised in this paper are demarcated in bold**

*For the first 16 items, please number each statement according to the identified scale of 0 to 100 (representing “I cannot do this at all” to “I am highly certain I can do this”)*

1. I can encourage everyone within the team to share ideas
2. I can read my friends’ and colleagues’ needs
3. **I can use clinical scenarios to achieve a deeper understanding of the basic science principles I have learned**
4. I can provide leadership within our team that creates an environment where things can be accomplished
5. I can recognize when someone wants comfort and emotional support, even if they do not overtly exhibit it
6. **I can apply the skills developed in CSI to evaluate other patient cases**
7. **I can find the information/resources needed for our team to do our job well**
8. I can recognize when a person is annoyed with me
9. **I can focus on individual patients in a holistic manner, incorporating elements of clinical and scientific significance**
10. When people experience a problem within our team, I can help to figure out what is going on
11. I can recognize when a person is inhibited by fear
12. **I can apply my understanding of basic science principles to clinical problems in order to contribute to better patient care**
13. **I can work with my team to achieve the goals we are set**
14. I can recognize when a person needs my help
15. **I can explain why clinical and basic science integrated teaching is important to my development as a doctor**
16. I can rely on the other people in my team to do their jobs well

*For items 17 to 19, please answer as openly and honestly as you can (free text)*

1. How has CSI helped in developing your ability to work effectively in teams?
2. How has CSI helped in developing your ability to build empathetic relationships with patients and peers / healthcare colleagues?
3. How has CSI helped in developing your ability to make connections between basic science and clinical medicine?
4. How has the integrated nature of CSI influenced your approach to learning?
