# Supplementary material for: Collaborative case-based learning with programmatic team-based assessment: a novel methodology for developing advanced skills in early-years medical students
Source: BMC Med Educ. 2022 Feb 7;22:81. doi: 10.1186/s12909-022-03111-5 (PMC8818362; doi:10.1186/s12909-022-03111-5)
Supplement: Supplementary file 2 — Additional file 2. [file 12909_2022_3111_MOESM2_ESM.docx]

## Additional File 2

## A selection of feedback comments relevant to the four core principles of CSI

| **CSI Core Principle** | **A Selection of Feedback Comments from surveys A, B and C** |
| --- | --- |
| Patient-centred learning | *It has made me think of patients [as] more than just a set of notes that you can follow; by understanding the patient's journey you are better able to care for them* |
|  | *Being able to look at a patient case from a scientific as well as human perspective has helped to really develop a holistic approach* |
|  | *Talking to people with differing understanding of concepts has reflected the variety in patients’ understanding of their illnesses* |
|  | *CSI really helps understand patient’s perspectives and their experiences of illness.* |
| Integration of clinical and scientific content | *[CSI] is one of the modules I've learned most in this year. Application and integration make you learn so much and you don't even realize it.* |
|  | *Through the face-to-face sessions, the teachers facilitate the connections, but later in the year I felt like I needed less help to make the same connections* |
|  | *CSI has helped me a lot - by linking the clinical cases to the basic science we have learnt in lectures, I find it far easier to remember and understand the science, as I can link it to real people* |
| Team-work and collaboration | *[Because] our team is quite varied, I have come to understand how to communicate and work with different people. Each person has their strengths, and acknowledging these has helped us work together better to get a task done* |
|  | *CSI is a good test of communication because [we] all have to agree and work together to find a solution. It also allows different viewpoints; every session someone has chipped in with an idea that no one else had thought of, which has started other brainstorms.* |
|  | *Time pressure means we're all willing to communicate effectively, so has greatly helped to increase this skill* |
|  | *I think we are still figuring out our group dynamic. We need to work on our time management, and try and share out the tasks equally, so that everyone is included (case 1)* |
|  | *All my efforts in trying to contribute have been dismissed (case 2)* |
| Motivating and engaging learning | *[It] was stimulating and mimicked how we will need to think as practicing doctors. I like that we get to test our knowledge in ways that we will actually use in the future - it has encouraged me to read around the material to understand how concepts are applied and prioritised in a clinical setting. I enjoy this style of assessment very much* |
|  | *The tAPP was really interesting; the combination of the medical history and access to the [resources] made it feel very similar to how I perceive clinical practice to be; problem solving, cross collaboration etc.* |
|  | *CSI is both fun and engaging. It is quite simply a medical student's playground to explore the wealth of information available to us, to make connections between basic science and clinical medicine, and to grow* |
|  | *Fast paced, no slides were provided afterwards, didn’t know what part I was supposed to make notes on (case 1)* |
|  | *Would be helpful if there could be more guidelines as different groups were taught different things (case 1)* |
